# Captopril reduces cardiac inflammatory markers in spontaneously hypertensive rats by inactivation of NF-kB

**DOI:** 10.1186/1476-9255-7-21

**Published:** 2010-05-12

**Authors:** José L Miguel-Carrasco, Sonia Zambrano, Antonio J Blanca, Alfonso Mate, Carmen M Vázquez

**Affiliations:** 1 Departamento de Fisiología y Zoología, Facultad de Farmacia, Universidad de Sevilla, E-41012 Sevilla, Spain

## Abstract

**Background:**

Captopril is an angiotensin-converting enzyme (ACE) inhibitor widely used in the treatment of arterial hypertension and cardiovascular diseases. Our objective was to study whether captopril is able to attenuate the cardiac inflammatory process associated with arterial hypertension.

**Methods:**

Left ventricle mRNA expression and plasma levels of pro-inflammatory (interleukin-1β (IL-1β) and IL-6) and anti-inflammatory (IL-10) cytokines, were measured in spontaneously hypertensive rats (SHR) and their control normotensive, Wistar-Kyoto (WKY) rats, with or without a 12-week treatment with captopril (80 mg/Kg/day; n = six animals per group). To understand the mechanisms involved in the effect of captopril, mRNA expression of ACE, angiotensin II type I receptor (AT1R) and p22phox (a subunit of NADPH oxidase), as well as NF-κB activation and expression, were measured in the left ventricle of these animals.

**Results:**

In SHR, the observed increases in blood pressures, heart rate, left ventricle relative weight, plasma levels and cardiac mRNA expression of IL-1β and IL-6, as well as the reductions in the plasma levels and in the cardiac mRNA expression of IL-10, were reversed after the treatment with captopril. Moreover, the mRNA expressions of ACE, AT1R and p22phox, which were enhanced in the left ventricle of SHR, were reduced to normal values after captopril treatment. Finally, SHR presented an elevated cardiac mRNA expression and activation of the transcription nuclear factor, NF-κB, accompanied by a reduced expression of its inhibitor, IκB; captopril administration corrected the observed changes in all these parameters.

**Conclusion:**

These findings show that captopril decreases the inflammation process in the left ventricle of hypertensive rats and suggest that NF-κB-driven inflammatory reactivity might be responsible for this effect through an inactivation of NF-κB-dependent pro-inflammatory factors.

## Background

The spontaneously hypertensive strain of the Wistar-Kyoto rat, that is the spontaneously hypertensive rat (SHR), provides a useful experimental model that develops hypertension and is characterized by structural and functional changes in the heart [1]. The heart to body mass ratio or the relative weight of the left ventricle, useful indices of ventricular hypertrophy at the organ level, have been found by several authors to be elevated in SHR [2-4]. This cardiac hypertrophy is correlated with an inflammatory process, suggesting that inflammation could be a key event in cardiovascular complications in hypertensive animals [5,6]. We and other authors have shown an increase in the heart inflammatory process in SHR [7], in L-NAME-induced arterial hypertension [8], as well as in hypertension induced by angiotensin II or aldosterone [9,10].

Angiotensin II (ANGII), the key effector of the renin-angiotensin system (RAS), plays an important role in the regulation of blood pressure, and there is accumulating evidence to indicate that ANGII is also capable of inducing an inflammatory response in the cardiac tissue through the activation of the nuclear factor κB (NF-κB) [11-13]. Recent studies have also described the role of interleukin-10 (IL-10) in ANGII-induced expression of pro-inflammatory cytokines and vascular dysfunction [14].

Angiotensin-converting enzyme (ACE) inhibitors have long been known to be effective in the reduction of blood pressure and in the regression of left ventricular hypertrophy both in humans with essential hypertension [15] and in different animal models of arterial hypertension, such as SHR [16] and NO-deficient hypertensive rats [17]. Moreover, the treatment with ACE inhibitors reversed the increase in the production of superoxide anions and the activation of NF-kB system, as well as the elevated expression of pro-inflammatory cytokines, in aortas of rat with reduced NO synthesis [18] and in a rabbit model of atherosclerosis [19]. Also, a reduction in the circulating levels of intercellular adhesion molecule-1 (ICAM-1) has been observed in hypertensive patients [20] and in human aortic endothelial cells [21] after the treatment with an ACE inhibitor.

Captopril is a widely used ACE inhibitor for treatment of arterial hypertension and cardiovascular diseases [22,23]. It has been shown to express immune regulating, antioxidant and anti-inflammatory properties [24]. Thus, captopril has been proved to be beneficial in experimental autoimmune encephalomyelitis [25], adriamycin-induced nephropathy [26], arthritis [27] and experimental rat colitis [28]. Captopril suppresses the inflammation in endotoxin-induced uveitis in rats [29]; improves oxidative stress in the kidney of L-NAME-treated rats [30], and increases the antioxidant defenses in mouse tissues [31]. In addition, the treatment with captopril produces a decrease in the left ventricle inflammatory cell infiltration in angiotensin II and aldosterone-salt-induced hypertension [32].

In the present work, we explore the effect of captopril on the cardiac inflammatory process associated with arterial hypertension. In particular, we have measured arterial blood pressure, heart rate, cardiac hypertrophy, as well as plasma levels and heart mRNA expression of pro-inflammatory (IL-1β and IL-6) and anti-inflammatory (IL-10) cytokines in SHR, with or without a chronic administration of captopril. In addition, the mRNA expression of ACE, angiotensin II type I receptor (AT1R), p22phox subunit of NADPH oxidase, and the expression and activation of NFκB, has also been measured in the left ventricles of these animals, in order to know the mechanism(s) involved in the anti-inflammatory effects of captopril.

## Methods

### Animals, treatments, heart tissue sampling and measurements in plasma

This study was conducted in accordance with the National Institutes of Health (NIH) Guide for the Care and Use of Laboratory Animals. Normotensive, male Wistar-Kyoto (WKY) and spontaneously hypertensive rats (SHR) aged 18-20 weeks were obtained from Harlan IBERICA, S.A. (Barcelona, Spain). Rats were housed at a temperature of 22-24°C in individual cages and freely fed a regular pellet diet. They were divided into four groups of six animals each: (1) WKY (control group), (2) SHR (untreated), (3) WKY treated with captopril (WKYCPT), and (4) SHR treated with captopril (SHRCPT). Captopril (CPT) was administered during 12 weeks as a dose of 80 mg/kg/day dissolved in the drinking water, the concentrations being adjusted according to daily water consumption and body weight in order to ensure correct dosage. Diastolic and systolic blood pressures, as well as the heart rate, were measured after the experimental period using the non-invasive and indirect method of tail-cuff occlusion in conscious animals using a NIPREM 645 pressure recorder (Cibertec, Barcelona, Spain). Body weight was determined on the same day that blood pressure was measured. At the end of the experimental period, all animals were fasted overnight before killing. They were anesthetized with pentobarbital (50 mg/kg i.p.) and blood samples were obtained by cardiac puncture and collected into tubes containing lithium heparin. Rats were killed by decapitation, then the heart was quickly removed and washed out with ice-cold 0.9% saline solution, and the left ventricle was dissected and weighed to calculate the relative left ventricle weight/body weight (LVW/BW). Samples of the left ventricle were frozen and stored at -70°C until use. To minimize diurnal variations, rats were routinely killed between 09:00 and 10:00 hours.

Plasma was separated by low-speed centrifugation at 1500 g and at 4°C for 30 min. IL-1β, IL-6 and IL-10 were measured in plasma using a quantitative sandwich enzyme immunoassay (commercial ELISA kits). Rat-specific monoclonal antibodies of each cytokine were pre-coated onto microplates (Pierce Biotechnology, Rockford, IL).

### NF-κB activity

Left ventricles from all experimental animal groups were used for determination of NF-kB activity. Nuclear extracts from left ventricles were obtained using commercially available nuclear extraction kits (Active Motif, Madrid, Spain) following the manufacturer's recommendations. P65 NF-κB activity was assessed with a transcription factor assay kit according to the manufacturer's instructions (TransAM NF-kB p65 kit, Active Motif, Madrid, Spain), which can measure the binding of activated p65 NF-kB to its consensus sequence attached to a microwell plate. Antibodies for the activated form of the p65 subunits of NF-κB were included in the assay.

### Reverse transcription and real-time polymerase chain reaction (RT-PCR)

Reverse transcription (RT) and real-time PCR was analyzed as previously reported [8]. Whole RNA was extracted from frozen left ventricle rats after homogenization with 1 mL of Tripure Isolation Reagent (Roche Diagnostics Corp., Indianapolis, USA) as described by Chomczynski and Sacchi [33]. RT was carried out in a final volume of 100 μL using a Ready-To-Go You-Prime First-Strand Beads (GE Healthcare, Madrid, Spain) according to the supplier's protocol. After RT, cDNA was purified using a commercial kit (GFX DNA purification kit, GE Healthcare, Madrid, Spain). cDNA was then diluted in sterile water and used as template for the amplification by the polymerase chain reaction (PCR). The specific mRNA sequences were amplified using the following pairs of primers: (from 5' to 3'):

IL-1β forward: GAGG CTGACAGACCCCAAAAGAT.

IL-1β reverse: GCACGAGGCATTTTTGTTGTTCA (product size 336 bp).

IL-6 forward: GAAATACAAAGAAATGATGGATGCT.

IL-6 reverse: TTCAAGATGAGTTGGATGGTCT (310 bp).

p22phox forward: GCTCATCTGTCTGCTGGAGTA,

p22phox reverse: ACGACCTCATCTGTCACTGGA (434 bp);

ACE forward: CAAAGTTCACTTGCTTCTTGG.

ACE reverse: TACTGTAAATGGTGCTCATGG (262 bp).

AT1R forward: CACCTATGTAAGATCGCTTC.

AT1R reverse: GCACAATCGCCATAATTATCC (445 bp).

p65 NF-κB forward: CCTAGCTTTCTCTGAACTGCAAA

p65 NF-κB reverse: GGGTCAGAGGCCAATAGAGA (71 bp).

IκB forward: TGGCTCATCGTAGGGAGTTT

IκB reverse: CTCGTCCTCGACTGAGAAGC (68 bp)

GAPDH forward: GCCAAAAGGGTCATCATCTCCGC.

GAPDH reverse: GGATGACCTGCCCACAGCCTTG (319 bp).

Each specific gene product was amplified by real-time PCR using Sybergreen TM reactions and an ABI PRISM 7000 Sequence Detection System (PE Applied Biosystems, Foster City, CA). Amplification data were collected by the sequence detector and analysed with sequence detection software. For each assay, a standard curve was constructed using increasing amounts of cDNA. In all cases, the slope of the curves indicated adequate PCR conditions (slope 3.2-3.4). The RNA concentration in each sample was determined from the threshold cycle (Ct) values and calculated with sequence detection software supplied by the manufacturer. The quantitative fold changes in mRNA expression were determined as relative to GAPDH mRNA levels in each corresponding group and calculated using the 2^-ΔΔCT ^method.

### Calculations and statistical analysis

The GraphPad Instat statistical program was used to analyze the data. All results were subjected to one-way analysis of variance (ANOVA) and represent means ± S.E.M. of six animals per experimental group. Differences in mean values between groups were assessed by Tukey-Kramer's test and considered statistically different at P < 0.05.

### Reagents

Unless otherwise specified, all reagents were obtained from Sigma Chemical (Madrid, Spain). Primers for RT-PCR analysis were synthesized by Tib Molbiol (Berlin, Germany). Captopril was obtained from Roig-Farma (Barcelona, Spain).

## Results

### Body weight, left ventricle weight, blood pressure and heart rate

No significant differences in body weights were observed among all four groups of animals at the end of the experimental period. The LVW/BW ratio (as an index of ventricular hypertrophy), as well as diastolic and systolic blood pressures and heart rate, were significantly increased in hypertensive animals (SHR) when compared with control, WKY rats. The treatment with captopril reversed these parameters back to normal values in SHR, and no effect of CPT was observed in WKY rats (Table 1).

**Table 1:** Final body weight, left ventricle weight/body weight ratio (LVW/BW), blood pressures, and heart rate in WKY, SHR, WKY treated with captopril (WKYCPT), and SHR treated with captopril (SHRCPT).

Parameter	WKY	SHR	WKYCPT	SHRCPT
Body weight (g)	391 ± 3	396 ± 10	372 ± 8	385 ± 16
LVW/BW (mg/g)	2.06 ± 0.08	2.68 ± 0.04***	1.93 ± 0.05^###^	2.01 ± 0.09^###^
Final DBP (mm Hg)	114 ± 1	207 ± 0.6***	115 ± 1^###^	117 ± 0.7^###^
Final SBP (mm Hg)	140 ± 0.9	231 ± 0.8***	139 ± 1.1^###^	138 ± 0.8 ^###^
Heart rate (beats/min)	342 ± 1.1	392 ± 2.6***	339 ± 1.8^###^	339 ± 1.7 ^###^

DBP, diastolic blood pressure; SBP, systolic blood pressure. Values are expressed as means ± SEM (n = 6). ***P < 0.001 vs. WKY; ^###^P < 0.001 vs. SHR.

### Plasma levels and cardiac mRNA expression of cytokines

**Figure 1:**
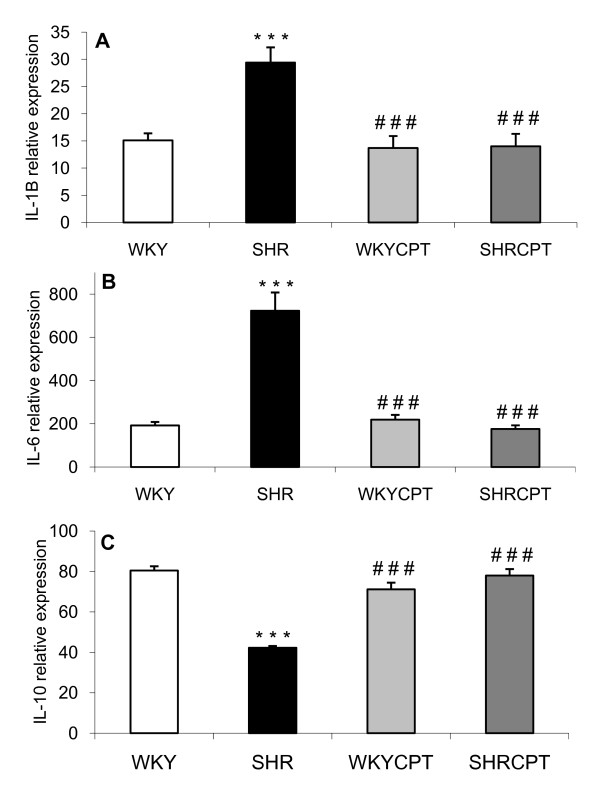
**RNA expression of interleukin-1β (A), interleukin-6 (B) and interleukin-10 (C) in WKY, SHR, WKY treated with captopril (WKYCPT) and SHR treated with captopril (SHRCPT)**. Values are expressed as means ± SEM (n = 6). The quantitative fold changes in mRNA expression were determined as relative to GAPDH mRNA levels in each corresponding group and calculated using the 2^-ΔΔCT ^method. ***P < 0.001 vs. WKY; ^###^P < 0.001 vs. SHR.

Plasma levels of IL-1β and IL-6 were significantly higher in SHR when compared with control, normotensive WKY rats (54% and 34%, respectively). The treatment with captopril was able to reverse these increases, reaching values similar to those observed in WKY rats. On the other hand, plasma levels of IL-10 were diminished in hypertensive rats (54%), this reduction being attenuated after treatment with captopril. No changes were observed between WKY and WKYCPT groups (Table 2). In addition, SHR showed a significant increase in the left ventricle mRNA expression of IL-1β and IL-6 together with a reduction in IL-10 when compared with WKY (15.1 ± 1.3 relative expression (RE) and 29.5 ± 2.8 RE for WKY and SHR, respectively, for IL-1β, p < 0.01; 192 ± 16.4 RE and 723 ± 85 RE for WKY and SHR, respectively, for IL-6, p < 0.001; 80.5 ± 2.1 RE and 42.3 ± 0.9 RE for WKY and SHR, respectively, for IL-10, p < 0.001). After the treatment with captopril, these changes were prevented and the mRNA expression in SHR became 13.7 ± 2.2 RE, 219 ± 22 RE and 71.2 ± 3.3 RE for IL-1β, IL-6 and IL-10, respectively. In contrast, no changes were observed in the expression of these cytokines in WKY rats treated with captopril (Figure 1).

**Table 2:** Plasma levels of interleukin-1β (IL-1β), interleukin-6 (IL-6) and interleukin-10 (IL-10) in WKY, SHR, WKY treated with captopril (WKYCPT), and SHR treated with captopril (SHRCPT).

Parameter	WKY	SHR	WKYCPT	SHRCPT
IL-1β (pg/mL)	19.8 ± 1.2	32.3 ± 1.7***	19.7 ± 0.8^###^	20.7 ± 1.8^###^
IL-6 (pg/mL)	81.7 ± 1	112.4 ± 7**	65.7 ± 1.6^###^	77 ± 6.5^###^
IL-10 (pg/mL)	57.5 ± 2.3	26.2 ± 0.9***	49.3 ± 1.7^###^	45 ± 3.3**^,###^

Values are expressed as means ± SEM (n = 6). **P < 0.01, ***P < 0.001 vs. WKY; ###P < 0.001 vs. SHR.

### Expression of ACE, AT1 receptor (AT1R) and p22phox in the heart

**Figure 2:**
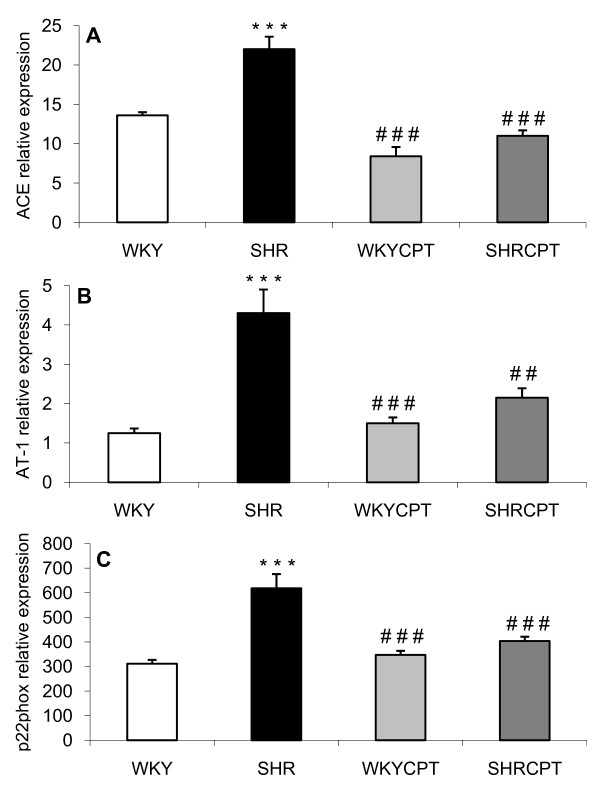
**RNA expression of angiotensin I converting enzyme, ACE (A), angiotensin II type I receptor, AT1R (B) and p22phox (C) in WKY, SHR, WKY treated with captopril (WKYCPT) and SHR treated with captopril (SHRCPT)**. Values are expressed as means ± SEM (n = 6). The quantitative fold changes in mRNA expression were determined as relative to GAPDH mRNA levels in each corresponding group and calculated using the 2^-ΔΔCT ^method. ***P < 0.001 vs. WKY; ^##^P < 0.01, ^###^P < 0.001 vs. SHR.

Figure 2 shows ACE, AT1R and p22phox mRNA expressions in left ventricles from all groups of rats. The expression of these tree molecules was significantly increased in SHR over control, WKY rats (13.6 ± 0.4 RE and 21.9 ± 1.6 RE for WKY and SHR, respectively, for ACE, p < 0.01; 1.3 ± 0.1 RE and 4.3 ± 0.6 RE for WKY and SHR, respectively, for AT1R, p < 0.001; 311 ± 16 RE and 618 ± 58 RE for WKY and SHR, respectively, for p22phox, p < 0.001). The administration of captopril to hypertensive rats was able to prevent the abnormally high values for these parameters and maintained their expressions at levels similar to those from the control group (10.9 ± 1 RE, 2.2 ± 0.3 RE and 403 ± 18 RE for ACE, AT1R and p22phox, respectively). No effects were observed in WKY rats treated with captopril.

### Heart mRNA expression of the system NF-κB/IκB

**Figure 3:**
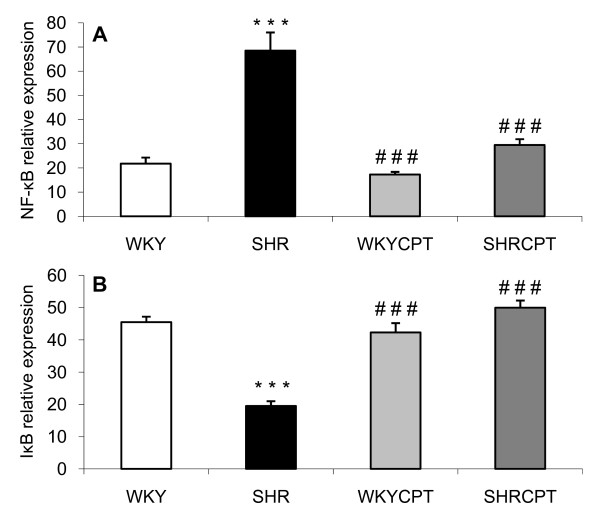
**RNA expression of NF-κB (A) and IκB (B) in WKY, SHR, WKY treated with captopril (WKYCPT) and SHR treated with captopril (SHRCPT)**. Values are expressed as means ± SEM (n = 6). The quantitative fold changes in mRNA expression were determined as relative to GAPDH mRNA levels in each corresponding group and calculated using the 2^-ΔΔCT ^method. ***P < 0.001 vs. WKY; ^###^P < 0.001 vs. SHR.

SHR showed a significant increase in the expression of NF-κB when compared with WKY rats (21.8 ± 2.5 RE and 68.5 ± 7.5 RE for WKY and SHR, respectively, p < 0.001), and captopril prevented this alteration (29.5 ± 2.4 RE for SHR treated with captopril). On the other hand, when mRNA expression of IβB was determined, a significant decrease was observed in hypertensive animals (45.5 ± 1.7 RE and 19.5 ± 1.5 RE, for WKY and SHR, respectively, p < 0.001), and the treatment of captopril was also able to inhibit this observation (50.0 ± 2.2 RE for SHR treated with captopril). Once again, no differences were observed between captopril-treated or untreated WKY rats (Figure 3).

### NF-κB activation

**Figure 4:**
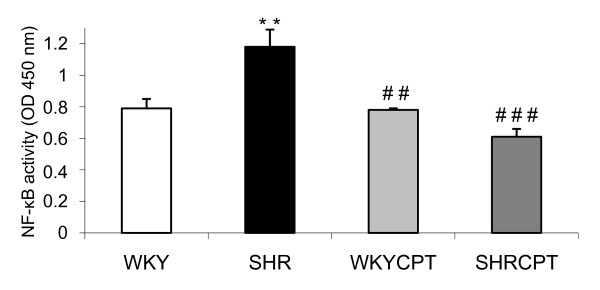
**Nuclear factor-kappa (NF-κB) activity in nuclear extract of the left ventricle in WKY, SHR, WKY treated with captopril (WKYCPT) and SHR treated with captopril (SHRCPT)**. Values are expressed as means ± SEM (n = 6). **P < 0.01 vs. WKY; ^##^P < 0.01, ^###^P < 0.001 vs. SHR.

We further investigated the effect of captopril on cardiac NF-κB activation. We found a 49% increase in left ventricle NF-κB p65 binding activity in SHR compared with WKY rats (0.79 ± 0.06 optical density (OD) and 1.18 ± 0.11 OD for WKY and SHR, respectively, p < 0.001). This activation was significantly inhibited in SHR treated with captopril (0.61 ± 0.05 OD for SHR treated with captopril). No changes were observed between WKY and WKY treated with captopril (Figure 4).

## Discussion

The aim of the present work was to explore the effect of captopril, a known ACE inhibitor, on the cardiovascular inflammatory process associated with arterial hypertension. In the current study we demonstrate, for the first time, that treatment with captopril reverses the observed increase in the gene expression of pro-inflammatory cytokines (IL-1β and IL-6), and the decrease in the gene expression of anti-inflammatory cytokines (IL-10), in the left ventricle of SHR; this effect is mediated by an inactivation of RAS that in turn produces an inhibition of NADPH oxidase and a reduced activation of NF-κB.

At the end of the treatment period, we observed an increase in blood pressures and in the heart rate in SHR when compared with WKY rats, as expected from previous observations [34-37]. The high levels of blood pressure and heart rate were accompanied by increases in the relative left ventricle weight and in plasma levels of both IL-1β and IL-6, and also by a reduction in the plasma levels of IL-10. An increase in circulating pro-inflammatory markers has been found in hypertensive patients [38] and in patients with pulmonary arterial hypertension [39], as well as in SHR [40], and in NO-deficient rats [8]. In contrast, a decrease in plasma levels of IL-10 has been found in patients with coronary artery disease and arterial hypertension [41], and in patients with pulmonary arterial hypertension [39]. In addition, IL-10 prevented the development of monocrotaline-induced pulmonary arterial hypertension in rats [42]. The observed changes in plasma levels of inflammatory markers in SHR were also accompanied by similar alterations in the left ventricle mRNA expression of these cytokines. These results are in agreement with previous studies showing that cardiac hypertrophy is associated with an inflammatory process in the myocardium of SHR [7,16] and other models of hypertension rats [8,17]. Moreover, the mRNA expression of IL-1β and IL-6 [40], intercellular/vascular cell adhesion molecules (ICAM, VCAM) and monocyte chemoattractant protein (MCP-1), have been reported to be enhanced in aortas of hypertensive rats [18,40], and in cardiac tissue from different rat models of hypertension [5,43]. However, to our knowledge, this is the first evidence concerning the decrease in plasma levels and left ventricle mRNA expression of IL-10 in hypertensive animals. Previous work has proven that endogenous IL-10 limits angiotensin II (ANG II)-mediated oxidative stress, inflammation and vascular dysfunction both in vivo and in vitro, suggesting a protective action of IL-10 in vascular diseases such as arterial hypertension [14]. In fact, IL-10 attenuates the increases in vascular superoxide and endothelial dysfunction during diabetes and atherosclerosis [44,45]. In the same way, it could be suggested that IL-10 might be a mediator of cardiac protection against arterial hypertension.

The administration of captopril was able to prevent the increase in blood pressures and heart rate observed in SHR and reversed the enhancement in the left ventricle weight index completely. These results are in agreement with previous studies using SHR [16,46], NO-deficient hypertensive rats [47] and obese Zucker rats, a model of non-insulin-dependent diabetic hypertensive rats [48]. In addition, when captopril was chronically administered, the observed alterations in hypertensive animals regarding plasma levels and left ventricle mRNA expression of inflammatory cytokines disappeared, the values reaching levels similar to those observed in WKY rats. In a previous work, enalapril (another ACE inhibitor) was able to produce an increase in plasma levels of IL-10 in patients with coronary artery disease and arterial hypertension[41]. Therefore, these results suggest an anti-inflammatory, cardioprotective effect of captopril in arterial hypertension, with a reduction in the circulating pro-inflammatory markers and an increase in those anti-inflammatory cytokines; these effects result in a benefit on the myocardial inflammatory process associated to arterial hypertension.

This anti-inflammatory effect of captopril has been previously proved in other diseases characterized by an increase in the inflammation process, such as nephropathy [26], arthritis [27], colitis [28] and uveitis [29]. Moreover, captopril produced a reduction in the inflammatory cell infiltration in the left ventricle of rats with angiotensin II and aldosterone-salt-induced hypertension [23,32].

Since several studies have shown the role of ANGII in the beneficial effect of captopril in arterial hypertension [30,49], more studies were designed in order to understand the mechanisms involved in the anti-inflammatory properties of captopril in the left ventricle of SHR. Our results indicate that captopril might act via interfering with NF-kB pathway activation, most probably by blocking the ANGII II production in the left ventricle of hypertensive animals. We found that captopril was able to prevent the increase in the left ventricle expression of ACE and AT1R observed in SHR, leading to a decrease in the local production and effect of ANGII. Bolterman et al. [34] previously observed an increase in ANGII levels in plasma of SHR when compared with WKY rats, which was corrected after the treatment with captopril. In addition, a specific down-regulation of ACE and AT1R by captopril has been also demonstrated in human dendritic cells [50] and in aorta and heart tissue of fructose-fed rats [51], respectively.

ANGII stimulates various signalling pathways that lead to NF-kB activation [11,52]. Thus, ANGII stimulates NADPH oxidase, which generates reactive oxygen species (ROS), and ROS are involved as second messengers in NF-κB activation and cytokine expression [53]. NF-κB plays a crucial role regulating at the transcriptional level several pro-inflammatory genes [6]. ANGII influences NF-κB activation by stimulating the nuclear translocation of the p65 subunit, DNA binding, transcription of a NF-κB reporter gene and I-κB degradation. Conversely, NF-κB stimulates the expression of the gene encoding angiotensinogen, thereby amplifying the ANGII-mediated inflammatory process [54]. On the other hand, it has been reported that a major role for IL-10 is to inhibit the expression of pro-inflammatory cytokines, such as IL-6, through an inactivation of NF-κB [55].

Numerous studies have shown that NF-kB participates in the vascular, renal and cardiac inflammatory processes in different models of arterial hypertension through its ability to activate a variety of inflammation-mediating genes. In experimental models of hypertension, the inhibition of NF-kB prevented ANGII-induced expression of IL-6, VCAM-1 and MCP-1, thus attenuating the inflammatory damage caused by ANGII [56,57].

The present study shows that the increase in the mRNA expression of IL-1β and IL-6 and the decrease in the mRNA expression of IL-10 in SHR is associated with an abnormally high cardiac mRNA expression of both p22phox (subunit of NADPH oxidase) and NF-κB, and a lower expression of IκB (a molecule that inhibits the translocation of NF-κB to the nucleus and its subsequent activation). In addition, a higher activation of NF-κB has been found in the left ventricle of hypertensive animals when compared with WKY rats. These results are in agreement with previous studies that reported an increase in NF-κB expression in SHR aorta and left ventricle [40,56], and also in the renal activation of NF-κB in SHR and in salt-sensitive hypertension [4,58].

When captopril was administered to hypertensive rats, the decrease in mRNA expression of pro-inflammatory markers and the increase in the expression of anti-inflammatory cytokine were accompanied by a reduction in the gene expression of p22phox and NF-κB, and by an increase in that of IκB. Moreover, captopril was able to reverse the activation of NF-κB found in left ventricle of SHR. These results suggest an inactivation of the NF-κB system in the beneficial effect of captopril in hypertension-induced cardiac damage. A decrease in the gene expression of NF-κB has been observed in aortas of SHR treated with the AT1R blocker, candesartan [59], and inactivation of NF-κB has been found in aortas of rats infused with ANGII and then treated with losartan [60]; in macrophages and vascular smooth muscle cells from a rabbit model of atherosclerosis after the treatment with quinapril [19]; in the kidney cortex of rats with unilateral urethral obstruction treated with enalapril [61], and in captopril-treated uveitis [29]. In contrast, no changes in the protein expression of NF-κB was reported in the left ventricle of captopril-treated SHR[4].

It has been proposed that an increased superoxide production contributes to the development of hypertension via activation of the sympathetic nervous system [62]. The presence of changes in heart rate in captopril-treated rats suggests that the effect of sympathetic function might play a major role in the antihypertensive effect of this compound. Moreover, the anti-inflammatory effect of captopril occurred together with a decrease in blood pressure. It has been demonstrated that mechanical stress induces reactive oxygen species formation via an up-regulation of NADPH oxidase [4,62]. Therefore, the observed anti-inflammatory and cardiac effects of captopril in SHR might be due not only to the inactivation of RAS above mentioned, but also to a reduction of the mechanical stress on the vessel wall.

## Conclusions

Our findings show that captopril decreases the inflammation process in the left ventricle of hypertensive rats through a reduction in the activation of NF-κB-dependent pro-inflammatory factors. Since the anti-inflammatory effect of captopril occurred together with a reduction in blood pressure and heart rate, it is possible that a part of the observed anti-inflammatory and cardioprotective effects of captopril might be due to the reduction in mechanical stress on the wall. Thus, it could be proposed that both the inactivation of RAS and blood pressure reduction are mechanisms accounting for the anti-inflammatory effect of captopril in SHR.

## Abbreviations

ACE: angiotensin converting enzyme; IL-1β: interleukin-1β; IL-6: interleukin-6; SHR: spontaneously hypertensive rats; WKY: Wistar Kyoto rats; AT-1R: angiotensin II type I receptor; ANG II: angiotensin II; RAS: renin-angiotensin system; NF-κB: nuclear factor κB; I-κB: nuclear factor κB inhibitor; NO: nitric oxide; ICAM-1: intercellular adhesion molecule-1; CPT: captopril; RT-PCR: reverse transcription and real time polymerase chain reaction; GAPDH: glyceraldehyde 3-phosphate dehydrogenase; NADPH: nicotinamide adenine dinucleotide phosphate.

## Competing interests

The authors declare that they have no competing interests.

## Authors' contributions

The manuscript was written and experiments designed by AM and CMV. All experiments were performed by JLMC, SZ and AJB, and supervised by AM and CMV. All authors have given final approval of the version to be published.
